# Pragmatics and Theory of Mind across cultures

**DOI:** 10.1098/rstb.2023.0500

**Published:** 2025-08-14

**Authors:** Mele Taumoepeau

**Affiliations:** ^1^ Te Herenga Waka, Victoria University of Wellington, Wellington 6140, New Zealand

**Keywords:** culture, theory of mind, parent–child interactions, social cognition

## Abstract

In this article, I present a cultural framing of pragmatic communication for understanding children’s developing understanding of their own and others’ minds. Taking a social constructivist approach, I reexamine evidence for the socialization of mental state understanding from a pragmatic standpoint. I pay particular attention to how cultural variation in caregivers' use of mental state language may reflect variation in pragmatic intentions that correspond with culturally framed socialization practices. I then consider how variation in the socialization of pragmatic intentions informs our understanding of culture-specific differences in theory of mind development. I conclude by suggesting that in diversifying the ways in which we think about communication and its role in making sense of the social world, we can begin to better understand the significance of children’s cultural context in this development.

This article is part of the theme issue ‘At the heart of human communication: new views on the complex relationship between pragmatics and Theory of Mind’.

## 1. Introduction

Communication and its role in inducting children into a ‘community of minds’ [1] is vital for children’s and young people’s ability to participate in and navigate their social worlds [2–4]. Pragmatics—the ability to use contextual information, including cultural norms and conventions to effectively communicate—invites particular attention to its use as a human tool for facilitating social understanding. Effective communication is not just the ability to understand and use words meaningfully and grammatically, but to interpret and convey our specific intentions. Consequently, pragmatic ability and its development are entwined with a suite of cognitive tools including formal language, mentalizing and executive functions [5]. Together, these mental tools support the cognitive and social capacity to attribute mental states to oneself and others. This is most evident in the development of advanced pragmatic skills, such as the interpretation of metaphor and irony requiring language users to actively seek out the intended meaning of the utterance that may be counterfactual to the literal meaning. As children enter middle childhood, their capacity to understand the figurative, metaphorical and ironic intentions of the speaker begins to develop [6,7], and recent findings indicate that higher levels of pragmatic understanding are predictive of better theory of mind [8–10], and that parental use and attitude towards the use of irony are related to children’s ability to comprehend speakers’ intended meaning [11]. Effective communication, however, takes place within a cultural organizing framework such that the knowledge one brings to the communicative exchange reflects patterns of cultural meaning accumulated through a range of communicative exchanges and cultural institutions [12]. Despite the emerging evidence of cultural differences in the development of these higher-level pragmatic skills [13,14], questions remain as to how early pragmatic development is integrated within a theory of social understanding, in such a way that draws on cultural knowledge patterns.

## 2. Socio-cultural theory

There is a long history of theory of mind theorizing within a socio-cultural framework, signalling a departure from the original sets of theory of mind theories that posit that children’s psychological life develops through abstract and individual reflections on the mind. Scholars began to consider how social experience and the relationships that are fostered through development are the source of children’s developmental progression in mind reading [1,15,16]. The significance of including social interaction at the centre of children’s development is that it brought to the fore two key ideas. First, that social understanding development moves from a purely individual cognitive endeavour to a socio-cognitive endeavour, centred in social interactions [15]. Second, language and communication form a conduit of cultural knowledge and a critical source of insight for how children learn about the mind during infancy and into the middle years [2,17]. This approach is thus ripe for a focused examination of the significance of culture in determining the role of pragmatic communication for understanding the developmental trajectories of social understanding across childhood.

What do we mean by culture, and how might it inform this analysis? Zooming out, cultural evolutionary approaches propose that mentalizing evolved through cultural learning—the learning of skills from others that are refined and evolved over time, which then transfer and contribute to cumulative culture [18]. In turn, mentalizing facilitates further learning through the cultural benefits it affords. Zooming in, social interaction as the source of children’s cultural learning is the mechanism for the transmission of ideas, skills and learning situated within cultural agreed-upon ways of being. Thus, the ways in which children are socialized reflect parental goals framed by specific cultural values that are then enacted through caregiver interactions with children [19–22]. It is the characteristics of this socialization process that raise some of the interesting questions regarding the specific role of language in the cultural ontogeny of mindreading. To what extent might this cultural mechanism have evolved in ways that serve particular cultural contexts or groups? How might culture contribute to and explain the construction of social understanding [23]?

## 3. Communication and Theory of Mind

Communication between individuals in groups is an immensely powerful tool for children’s cultural learning. Indeed, Heyes & Frith [24] suggest that the process of acquiring an understanding of mind takes place through effortful work that is driven by language and represents cultural learning. From a Vygotskian perspective, conversations are a social context in which references to mental states in the context of actions and events allow children to first experience these words and actions between social partners, before internalizing these concepts [25]. Consequently, extensive work has focused on such language exchanges that occur between family members, either in mother–child dyads or in family and sibling groups [26–32].

Particular interest has been in caregiver references to mental state terms because they provide tangible labels for organizing and structuring actions and behaviour that may share common underlying internal and mental motivations and attributions [33]. For example, reaching for a piece of fruit, conveying an expression of joy at seeing a doughnut, and cuddling a toy, all share an underlying mental state of desire and can be labelled as such. Children also learn that the experiences underlying a mental state may differ from person to person—someone may express joy at eating hokey pokey icecream, but such joy may not be shared by another person. Thus, directly labelling these mental states can play an important role in drawing attention to the unseen contents of mind and thereby help children reflect and internalize the understanding that people may share different attitudes towards the same event. Indeed, evidence from studies across childhood has offered compelling support that theory of mind is socialized through conversations about mental states with a range of social partners within family [28,32–40] and school environments [41,42], including causal evidence that discussions about mental states in older childhood facilitate ToM understanding by increasing the accuracy of mental state attributions [43].

## 4. Pragmatics and Theory of Mind

Despite the well-established connection between social partners’ explicit references to mental state terms and children’s developing understanding of mental states, others take a different view of the significance of such terms, calling for greater attention to broader features of communication [44–46]. A broader approach to communication *per se* may help further explain variance in children’s developing social understanding beyond that which can be accounted for by the socialization of mental state language. Relatedly, as I will address later, this approach may also offer instructive direction for understanding why there is cross-cultural variation in developmental trajectories of social understanding, such as culturally linked differences in the developmental sequencing of children’s understanding of knowledge access versus diversity of belief [47].

Proponents of process-interactional approaches take routine activity and interaction as the focal starting points for social development, centred in meaningful relationships [15,48–50]. The role of communication on this account is to help the child understand how their engagement with the world can be explained or understood in psychological terms [46,51]. Relatedly, more specific attention to the pragmatic features of conversation as a conduit of mental state understanding has been proposed by Harris [45], who posited that the pragmatics of conversations irrespective of the use of mental state verbs is as important for helping children understand the multiple perspectives on an event. Holding a conversation by its very nature will involve the introduction and elaboration on multiple perspectives [52].

From a pragmatic perspective, understanding a conversation requires more than simply decoding the syntactic and semantic form of the utterances, but critically, it also requires interpretation of the *intended* meaning of the utterances [53]. Correctly interpreting the intended meaning, however, involves drawing on a range of contextual information and assumptions to make sense of the linguistic and referential ambiguities. A Gricean approach to understanding how we do this has centred around the notion of the Cooperative Principle—that our conversations, rather than a set of disconnected utterances, involve the active cooperation between conversational interlocutors to arrive at an understanding, through an iterative series of conversational exchanges. To achieve this, Grice proposed four maxims that together underlie the cooperative principle. Do not say more or less than you need to (Quantity), do not say what you believe to be false, or that you lack evidence for (Quality); be relevant (Relevance) and avoid ambiguity or obscurity of expression, or unnecessary words (Manner). It is when a listener understands that there has been some violation of one or more of these maxims that the inferential work takes place to uncover the intended meaning [54].

Relatedly, utterances do more than simply describe a state of affairs of the world, but rather perform acts that convey specific intended meaning that must be interpreted by the listener. Speech Act theory has been concerned with understanding the conditions under which a litener may correctly infer the speaker's underlying intention [55,56]. Much of what we say to each other in these conversational contexts requires the listener to recognize what the speaker’s intentions are in performing a specific speech act. Thus, the very skills that mature listeners and speakers require to understand and convey intended meaning recruit socio-cognitive knowledge [57]. Consequently, it becomes difficult to disentangle pragmatic language skill from social understanding, particularly in early development [58]. However, in a highly informative study with younger children who may not yet have acquired complex pragmatic understanding of language, there are clear indications that training children to attend to the discourse features of a conversation—making explicit different points of view—increased their success on theory of mind tasks [59]. Performance in the discourse condition was on a par with the condition that specifically focused on the deceptive nature of the task, or mental states. Importantly, training children to attend to both the discourse features of the condition, as well as the psychological content of the task, yielded the most successful outcome [59].

## 5. Cultural socialization of pragmatic communication

A consideration of the pragmatic requirements of conversational exchanges is consistent with a socio-cultural approach that emphasizes a co-construction of meaning through social exchanges in which the child is an active partner [1,15,48,60]. Common ground—the shared assumptions and knowledge that one brings to the exchange—is crucial for how we verbally and non-verbally monitor and manage our conversational interactions [61]. Building this shared understanding is established early in ontogeny through the development of children’s joint attention and pointing, which provide explicit contexts for caregivers to modify and tailor interactions with children on the basis of their common ground. These early forms of engagement provide a foundation for children to develop an orientation towards the world in which others have a perspective on the infant’s actions [62]. The two-step proposal of O’Madagain & Tomasello [63] links joint attention to objects with later joint attention to mental contents, which require both coordination towards mental contents and the understanding of differences in our attitudes or perspectives towards that mental content.

At a verbal level, conversational exchanges, and in particular the use of sentential complement clauses, are a vehicle through which the appreciation of third-person perspectives may develop. Although references to mental states are an explicit way of introducing perspective in discourse, it is however becoming increasingly clear that there is cultural variation in the extent to which mental state terms are evidenced in parent–child conversations. This tendency likely reflects a particular cultural perspective in which psychological autonomy is fostered through references to the child’s own goals, intentions and mental states [64] and evidenced through treating the child as an equal conversational partner [12]. For instance, in a within-culture study, Taumoepeau [65] showed that variation in the tendency for Pacific families living in New Zealand to refer to mental states was associated with the extent to which they identified with their cultural membership, with more European-oriented families using more cognitive terms then more Pacific-oriented families. In cultural contexts where collective values are more central, parents place relatively more importance on talking about behaviours [66,67], rules and social norms [68] and actions [69] rather than mental states. The variation in what parents choose to talk about accords with particular cultural patterns of socialization and the values and beliefs underpinning this socialization.

Against this background, Ilgaz & Allen [23] argue for broadening the scope under which parent–child interactions are coded for perspectival intent. Observational findings indicate that mothers may help children understand the multiple perspectives involved in the communicative exchange. Using book reading as an example, Adrian *et al*. [70] argued that the nature of book reading contexts exposes children to multiple viewpoints—that of the story, of the parent and of the child—and that this is an opportunity to explore these perspectives. The communicative intentions expressed during the storybook reading process are designed to help the mother to organize her discourse through a variety of pragmatic functions. For instance, various speech acts organize the range of mental view points—that of the character, of the child and of the parent.

Although the structure of conversational encounters may help children see how multiple perspectives can be negotiated, these forms of communicative exchanges are also culturally mediated and assume particular socialization goals, norms and expectations [71–73]. For instance, cultural values of hierarchy versus egalitarianism are reinforced in the communicative intentions underlying three forms of speech acts: requests for information (e.g. questions), assertives (statements or comments) and requests for action (imperatives, commands and prescriptives) [74]. The focus on cognitive development reflects an emphasis on psychological autonomy [75,76] and is evident in the high use of questions and assertives that draw attention to the mental state of the listener. Although the type of question varies in its engagement with the particular mental attitude of the listener, the tendency to question may reflect a developmental scaffolding of inquiry into the mind. Similarly, assertives—responses to questions or elaborations on the caregiver’s or the child’s utterances—are more evident in contexts where children are treated as quasi-equal conversational partners and reflect particular cultural attitudes towards language socialization [74]. For instance, in a comparative study of language socialization in naturalistic environments of Dutch and urban and rural Mozambique families, Dutch caregivers were more likely to engage in utterances that signal cognitive intentions by referring to contextually salient information such as object labelling, or requests for such information [77]. In contrast, in the Mozambique families who favour support for community through social responsibilities and where hierarchical norms are important, there were higher instances of imperatives—utterances with clear instructions related to physical activities and that elicit clear and unambiguous action-related behaviour. The high frequency of imperatives is evident in a range of cultural groups that endorse similar cultural mandates of action autonomy such as in Sub-Saharan Africa [74,78] and Guatemala [79].

Cultural variation in the use of repetitions in language interactions reveals preferences for pragmatic intentions that accord with specific cultural socialization goals [12,80]. The socialization of language through a form of repetition known as *expansions* or *recasting* is a particularly interesting case in point. In cultural groups where psychological autonomy is encouraged, child-directed language is modified to allow children to participate as equal conversational partners. This modification can take place in the form of expansion utterances where a more competent communicator elaborates and expands on a child’s platform utterance in a more syntactically complex way [81,82]. Pragmatically, however, expansions offer a mechanism for caregivers to elucidate the intended meaning of the child, by presenting an alternative utterance that may more accurately reflect what the child is intending to communicate. By attempting to ground the child’s utterance, meaning is established. Yet, as Ochs & Schieffelin [12] articulate, there are particular assumptions inherent in this type of exchange such that the child has a particular perspective on an event, and that the mother reinforces this through her attempts to clarify the child’s utterance. Thus, the preference for this type of exchange may be especially important for building an appreciation of multiple perspectives and may foster particular understanding in diversity of belief.

In cultural contexts that foster social integration and interdependent self-development, language socialization is encouraged through children’s repetition of the caregiver’s utterances. The function of these types of repetition is to emphasize an outward orientation towards others, such as learning how to adapt and respond in verbal and non-verbal ways in different social contexts (see [80] for review). *Prompting* the child to exactly repeat the parents’ utterance to a third party or instructing the child on what to say in a particular situation through *guided repetition* emphasizes the transfer of knowledge. These types of language interactions are consistent with social learning approaches that value acquiring knowledge and skill mastery through observation and faithful imitation [83,84].

## 6. Pragmatic communication and cultural effects in Theory of Mind

To what extent however, might cultural variation in language socialization patterns explain variation in children’s mentalizing ability? The few studies that test broader measures of parental cultural orientation or values—collectivism or individualism—do not appear to relate to developmental sequences in children’s developing theory of mind (e.g. [85,86]). However, these measures of cultural values may not be nuanced enough to capture the actual variation in language interaction style. There is some evidence that caregivers’ tendency to refer to mental states when interacting with their children can account for differences in the patterns of development in children’s mental state knowledge between two cultural groups. For instance, Taumoepeau *et al*. [68] showed that variation in false-belief performance between New Zealand and Iranian children was mediated by parents’ use of mental state talk. In Spanish contexts, parents use of emotion language was related to false-belief understanding, unlike in other settings where these links were not found [34]. Moreover, parents also engage with their children in mind-minded ways—treating them as agents with minds [87,88]. This style of talk has also been shown to account for cultural differences between Hong Kong and UK children’s theory of mind performance [89].

Language that draws attention to variation in viewpoints by making explicit the referent of the mental state has been shown to play a role in scaffolding mental state understanding [23,39,40,70]. In Western contexts, references to the *child’s* desires are initially more important for scaffolding children’s understanding of others’ emotional states, whereas later discussion about *others’* cognitions facilitates cognitive understanding [39,40]. The ways in which parents may choose to focus on the child’s or others’ mental states may reflect sensitivity on the part of the parents regarding their children’s level of understanding, which may provide a way of arriving at common ground and helping the child participate as a conversational partner. Importantly, referent use varies as a function of cultural group. As described above, in cultural contexts where harmony and interdependence are highly valued, caregivers are more likely to refer to others’ behaviours and actions to help orient children towards others. In these groups, caregivers are also more likely to focus on others’ mental states versus the child’s mental states and it is these other-oriented (rather than child-oriented) mental states that foster theory of mind in these cultural contexts [68,90].

There is also evidence, however, that other forms of linguistic interaction facilitate mental state understanding in ways that are consistent with cultural mandates. For instance, in Asian contexts, Japanese children use behaviours and rules to justify the protagonist’s actions [91] and drawing Chinese children’s attention to behaviours through clarifying causal and comparative explanations and descriptions, rather than mental states, was associated with false-belief understanding [92]. The authors propose that in cultural contexts where mental state discussions with children are rare, attention to behavioural cues is a way in which children can implicitly make links between behaviours and internal states.

Given the variation in the use of mental state language, a pragmatic approach may therefore be a fruitful avenue for exploring the cultural effects of communication style on the developmental sequencing of children’s mentalizing capacity. There are intriguing cultural differences in the sequencing of stages of theory-of-mind development that call for a closer examination of the socio-cultural contexts in which children are socialized (see [47,93,94]). One of the most well-documented differences is the ordering of children’s understanding of the source of knowledge and diversity of belief. Children from China, Iran, Vanuatu, Tonga and Israeli-Arab minority contexts show an advantage in knowledge access tasks over diverse-beliefs tasks [85,95–98], which is opposite to the developmental progression of children from European–American contexts [99]. Alongside distal explanations of influence such as level of schooling and socio-economic status, consideration of cross-cultural variation in pragmatic intentions in parent–child interactions may help elucidate the timing differences in theory-of-mind milestones. As illustrated above, pragmatic intentions vary as a function of cultural socialization goals, which may orient children’s attention to particular mental states over others and contribute to these differences in developmental timing. For instance, the focus on communicative acts, such as expansions, may help draw children’s attention to their own belief states, whereas acts that encourage repetition of knowledgeable speakers’ utterances focus on knowledge transfer and knowledge acquisition. Moreover, the other-orientation of parent conversational style, through the repetition of utterances, and the tendency to refer to *others’* mental states may also contribute to children’s ability to attend to knowledge mental states earlier. Indeed, Taumoepeau *et al*. [68] showed that when mothers focused more on their own, rather than the child’s mental states in a picture description task, this was associated with children who performed better in the knowledge access task relative to the diverse beliefs task. More work is needed, however, to directly address the relation between variation in pragmatic features and children’s theory of mind development.

In sum, paying closer attention to how pragmatic intentions drive the types of conversations that children engage in may reveal communicative profiles that foster culture-specific ways in which children make sense of their social interactions. In doing so, children’s attention is drawn to features of social life, and to explanations for social behaviour that foster different skills and abilities. By diversifying the ways in which we think about communication and its role in making sense of the social world, we can begin to better understand the significance of children’s cultural context in this development.

## Data Availability

This article has no additional data.

## References

[1] Nelson K. 2003 Entering a community of minds: an experiential approach to ‘theory of mind’. Hum. Dev. **46**, 24–46. (10.1159/000067779)

[2] Astington J, Baird J. 2005 Why language matters for theory of mind. Oxford, UK: Oxford University Press. (10.1093/acprof:oso/9780195159912.001.0001)

[3] Devine RT, Hughes C. 2016 Measuring theory of mind across middle childhood: reliability and validity of the silent films and strange stories tasks. J. Exp. Child Psychol. **149**, 23–40. (10.1016/j.jecp.2015.07.011) 26255713

[4] Osterhaus C, Bosacki SL. 2022 Looking for the lighthouse: a systematic review of advanced theory-of-mind tests beyond preschool. Dev. Rev. **64**, 101021. (10.1016/j.dr.2022.101021)

[5] Matthews D, Biney H, Abbot-Smith K. 2018 Individual differences in children’s pragmatic ability: a review of associations with formal language, social cognition, and executive functions. Lang. Learn. Dev. **14**, 186–223. (10.1080/15475441.2018.1455584)

[6] Lecce S, Ronchi L, Del sette P, Bischetti L, Bambini V. 2019 Interpreting physical and mental metaphors: is theory of mind associated with pragmatics in middle childhood? J. Child Lang. **46**, 393–407. (10.1017/s030500091800048x) 30442207

[7] Pouscoulous N. 2023 More than one path to pragmatics? Insights from children’s grasp of implicit, figurative and ironical meaning. Cognition **240**, 105531. (10.1016/j.cognition.2023.105531) 37611331

[8] Del Sette P, Bambini V, Bischetti L, Lecce S. 2020 Longitudinal associations between theory of mind and metaphor understanding during middle childhood. Cogn. Dev. **56**, 100958. (10.1016/j.cogdev.2020.100958)

[9] Lee K, Sidhu DM, Pexman PM. 2021 Teaching sarcasm: evaluating metapragmatic training for typically developing children. Can. J. Exp. Psychol. / Rev. Can. De Psychol. Expérimentale **75**, 139–145. (10.1037/cep0000228) 32757569

[10] Del Sette P, Bambini V, Tonini E, Lecce S. 2024 Are theory of mind and metaphor comprehension causally related? A training study in middle childhood. Lang. Acquis 1–21. (10.1080/10489223.2024.2345324)

[11] Banasik-Jemielniak N *et al*. 2020 "Wonderful! We’ve just missed the bus." - Parental use of irony and children’s irony comprehension. PLoS One **15**, e0228538. (10.1371/journal.pone.0228538) 32084153 PMC7034895

[12] Ochs E, Schieffelin B. 1984 Language acquisition and socialization: three developmental stories and their implications. In Culture theory: essays on mind, self and emotion (eds R Shweder, R Levine), pp. 276–320. Cambridge, UK: Cambridge University Press.

[13] Filippova E, Astington JW. 2008 Further development in social reasoning revealed in discourse irony understanding. Child Dev. **79**, 126–138. (10.1111/j.1467-8624.2007.01115.x) 18269513

[14] Shahaeian A, Nielsen M, Peterson CC, Slaughter V. 2014 Cultural and family influences on children’s theory of mind development: a comparison of Australian and Iranian school-age children. J. Cross Cult. Psychol. **45**, 555–568. (10.1177/0022022113513921)

[15] Carpendale JIM, Lewis M. 2004 Constructing an understanding of mind: the development of children’s social understanding within social interaction. Behav. Brain Sci. **27**, 79–151. (10.1017/s0140525x04000032) 15481944

[16] Rogoff B. 1990 Apprenticeship in thinking. New York, NY: Oxford University Press.

[17] Milligan K, Astington JW, Dack LA. 2007 Language and theory of mind: meta-analysis of the relation between language ability and false-belief understanding. Child Dev. **78**, 622–646. (10.1111/j.1467-8624.2007.01018.x) 17381794

[18] Heyes C. 2017 When does social learning become cultural learning? Dev. Sci. **20**, e12350. (10.1111/desc.12350) 26547886

[19] Keller H. 2003 Socialization for competence: cultural models of infancy. Hum. Dev. **46**, 288–311. (10.1159/000071937)

[20] Markus HR, Kitayama S. 1991 Culture and the self: implications for cognition, emotion, and motivation. Psychol. Rev. **98**, 224–253. (10.1037//0033-295x.98.2.224)

[21] Markus HR, Kitayama S. 2010 Cultures and selves: a cycle of mutual constitution. Perspect. Psychol. Sci. **5**, 420–430. (10.1177/1745691610375557) 26162188

[22] Super CM, Harkness S. 1986 The developmental niche: a conceptualization at the interface of child and culture. Int. J. Behav. Dev. **9**, 545–569. (10.1177/016502548600900409)

[23] Ilgaz H, Allen JWP. 2021 (Co-)constructing a theory of mind: from language or through language? Synthese **198**, 8463–8484. (10.1007/s11229-020-02581-8)

[24] Heyes CM, Frith CD. 2014 The cultural evolution of mind reading. Science **344**, 1243091. (10.1126/science.1243091) 24948740

[25] Vygotsky L. 1978 Mind in society: the development of higher psychological processes. Cambridge, MA: Harvard University Press.

[26] Brown JR, Dunn J. 1992 Talk with your mother or your sibling? Developmental changes in early family conversations about feelings. Child Dev. **63**, 336–349. (10.1111/j.1467-8624.1992.tb01631.x) 1611938

[27] Cutting A, Dunn J. 2006 Conversations with siblings and with friends: links between relationship quality and social understanding. Br. J. Dev. Psychol **24**, 73–87. (10.1348/026151005X70337)

[28] Devine RT, Hughes C. 2018 Family correlates of false belief understanding in early childhood: a meta‐analysis. Child Dev. **89**, 971–987. (10.1111/cdev.12682) 27883189

[29] Dunn J. 1999 Making sense of the social world: mindreading, emotion, and relationships. In Developing theories of intention (eds P Zelazo, J Astington, D Olson), pp. 229–242. New York, NY: Psychology Press. (10.4324/9781003417927-15)

[30] Dunn J, Brown J, Beardsall L. 1991 Family talk about feeling states and children’s later understanding of others’ emotions. Dev. Psychol. **27**, 448–455. (10.1037//0012-1649.27.3.448)

[31] Slomkowski C, Dunn J. 1996 Young children’s understanding of other people’s beliefs and feelings and their connected communication with friends. Dev. Psychol. **32**, 442–447. (10.1037//0012-1649.32.3.442)

[32] Tompkins V, Benigno JP, Kiger Lee B, Wright BM. 2018 The relation between parents’ mental state talk and children’s social understanding: a meta‐analysis. Soc. Dev. **27**, 223–246. (10.1111/sode.12280)

[33] Ruffman T, Puri A, Galloway O, Su J, Taumoepeau M. 2018 Variety in parental use of ‘want’ relates to subsequent growth in children’s theory of mind. Dev. Psychol. **54**, 677–688. (10.1037/dev0000459) 29154654

[34] Adrian JE, Clemente RA, Villanueva L, Rieffe C. 2005 Parent–child picture-book reading, mothers’ mental state language and children’s theory of mind. J. Child Lang. **32**, 673–686. (10.1017/s0305000905006963) 16220639

[35] Peterson C, Slaughter V. 2003 Opening windows into the mind: mothers’ preferences for mental state explanations and children’s theory of mind. Cogn. Dev. **18**, 399–429. (10.1016/s0885-2014(03)00041-8)

[36] Ruffman T, Slade L, Crowe E. 2002 The relation between children’s and mothers’ mental state language and theory‐of‐mind understanding. Child Dev. **73**, 734–751. (10.1111/1467-8624.00435) 12038548

[37] Sehlstedt I, Hansson I, Hjelmquist E. 2024 The longitudinal relations between mental state talk and theory of mind. BMC Psychol. **12**, 191. (10.1186/s40359-024-01692-y) 38582883 PMC10998333

[38] Symons DK. 2004 Mental state discourse, theory of mind, and the internalization of self–other understanding. Dev. Rev. **24**, 159–188. (10.1016/j.dr.2004.03.001)

[39] Taumoepeau M, Ruffman T. 2006 Mother and infant talk about mental states relates to desire language and emotion understanding. Child Dev. **77**, 465–481. (10.1111/j.1467-8624.2006.00882.x) 16611184

[40] Taumoepeau M, Ruffman T. 2008 Stepping stones to others’ minds: maternal talk relates to child mental state language and emotion understanding at 15, 24, and 33 months. Child Dev. **79**, 284–302. (10.1111/j.1467-8624.2007.01126.x) 18366424

[41] Lecce S, Ronchi L, Devine RT. 2022 Mind what teacher says: teachers’ propensity for mental‐state language and children’s theory of mind in middle childhood. Soc. Dev. **31**, 303–318. (10.1111/sode.12552)

[42] Lecce S, Ronchi L, Devine RT. 2024 The effect of peers’ theory of mind on children’s own theory of mind development: a longitudinal study in middle childhood and early adolescence. Dev. Psychol. **60**, 1269–1278. (10.1037/dev0001758) 38647470

[43] Bianco F, Lecce S, Banerjee R. 2016 Conversations about mental states and theory of mind development during middle childhood: a training study. J. Exp. Child Psychol. **149**, 41–61. (10.1016/j.jecp.2015.11.006) 26723472

[44] Harris P. 1996 Desires, beliefs, and language. In Theories of theories of mind (eds P Curruthers, P Smith), pp. 200–220. Cambridge, UK: Cambridge University Press. (10.1017/CBO9780511597985.014)

[45] Harris P. 1999 Acquiring the art of conversation: children’s developing conception of their conversational partner. In Developmental psychology: achievements and prospects (ed. M Bennet), pp. 89–105. Philadelphia, PA: Psychology Press.

[46] Turnbull W, Carpendale JIM. 2009 Talk and children’s understanding of mind. J. Conscious. Stud **16**, 140–166.

[47] Yu CL, Wellman HM. 2024 A meta-analysis of sequences in theory-of-mind understandings: theory of mind scale findings across different cultural contexts. Dev. Rev. **74**, 101162. (10.1016/j.dr.2024.101162)

[48] Nelson K. 1996 Language in cognitive development: the emergence of the mediated mind. Cambridge, UK: Cambridge University Press. (10.1017/CBO9781139174619)

[49] Nelson K. 2005 Language pathways into the community of minds. In Why language matters for theory of mind (eds J Astington, J Baird), pp. 26–49. Oxford, UK: Oxford University Press.

[50] Rogoff B, Mistry J, Göncü A, Mosier C. 1993 Guided participation in cultural activity by toddlers and caregivers. Monogr. Soc. Res. Child Dev. **58**, 1–174. (10.1111/1540-5834.ep11871652) 8284000

[51] Turnbull W, Carpendale JIM, Racine TP. 2008 Relations between mother-child talk and 3- to 5-year-old children’s understanding of belief: beyond mental state terms to talk about the mind. Merrill Palmer Q. **54**, 367–385. (10.1353/mpq.0.0004)

[52] Harris P, de Rosnay M, Pons F. 2005 Language and children’s understanding of mental states. Curr. Dir. Psychol. Sci. **14**, 69–73. (10.1111/j.0963-7214.2005.00337.x)

[53] Sperber D, Wilson D. 1986 Relevance: communication and cognition. Oxford, UK: Blackwell.

[54] Grice H. 1975, 1989 Logic and conversation. In Syntax and semantics 3: speech acts (eds P Cole, JL Morgan), pp. 41–58. New York, NY: Academic Press.

[55] Austin J. 1962 How to do things with words. Cambridge, MA: Harvard University Press.

[56] Searle J. 1969 Speech acts. Cambridge, UK: Cambridge University press.

[57] Rubio-Fernandez P. 2023 Cultural evolutionary pragmatics: investigating the codevelopment and coevolution of language and social cognition. Psychol. Rev. **131**, 18–35. (10.1037/rev0000423) 36862451

[58] Zufferey S. 2010 Lexical pragmatics and theory of mind: the acquisition of connectives. Amsterdam, The Netherlands: John Benjamins Publishing Company. See http://ebookcentral.proquest.com/lib/vuw/detail.action?docID=623371.

[59] Lohmann H, Tomasello M. 2003 The role of language in the development of false belief understanding: a training study. Child Dev. **74**, 1130–1144. (10.1111/1467-8624.00597) 12938709

[60] Carpendale J, Lewis C. 2006 How children develop social understanding. Oxford, UK: Blackwell Publishing.

[61] Clark EV. 2015 Common ground. In The handbook of language emergence (eds B MacWhinney, W O’Grady), pp. 328–353. Oxford, UK: John Wiley & Sons. (10.1002/9781118346136.ch15)

[62] Carpendale JIM, Carpendale AB. 2010 The development of pointing: from personal directedness to interpersonal direction. Hum. Dev. **53**, 110–126. (10.1159/000315168)

[63] O’Madagain C, Tomasello M. 2021 Joint attention to mental content and the social origin of reasoning. Synthese **198**, 4057–4078. (10.1007/s11229-019-02327-1)

[64] Lillard A. 1998 Ethnopsychologies: cultural variations in theories of mind. Psychol. Bull. **123**, 3–32. (10.1037//0033-2909.123.1.3) 9461850

[65] Taumoepeau M. 2015 From talk to thought: strength of ethnic identity and caregiver mental state talk predict social understanding in preschoolers. J. Cross Cult. Psychol. **46**, 1169–1190. (10.1177/0022022115604393)

[66] Doan SN, Wang Q. 2010 Maternal discussions of mental states and behaviors: relations to emotion situation knowledge in European American and immigrant Chinese children. Child Dev. **81**, 1490–1503. (10.1111/j.1467-8624.2010.01487.x) 20840236 PMC2956600

[67] Doan SN, Lee HY, Wang Q. 2019 Maternal mental state language is associated with trajectories of Chinese immigrant children’s emotion situation knowledge. Int. J. Behav. Dev. **43**, 43–52. (10.1177/0165025418783271)

[68] Taumoepeau M, Sadeghi S, Nobilo A. 2019 Cross-cultural differences in children’s theory of mind in Iran and New Zealand: the role of caregiver mental state talk. Cogn. Dev. **51**, 32–45. (10.1016/j.cogdev.2019.05.004)

[69] Cebioğlu S, Marin KA, Broesch T. 2022 Variation in caregivers’ references to their toddlers: Child-directed speech in Vanuatu and Canada. Child Dev. **93**, e622–e638. (10.1111/cdev.13833) 36062549

[70] Adrián JE, Clemente RA, Villanueva L. 2007 Mothers’ use of cognitive state verbs in picture‐book reading and the development of children’s understanding of mind: a longitudinal study. Child Dev. **78**, 1052–1067. (10.1111/j.1467-8624.2007.01052.x) 17650125

[71] Harkness S, Super CM (eds). 1996 Parents’ cultural belief systems: their origins, expressions, and consequences. New York, NY: Guildford Press.

[72] Rowe ML. 2008 Child-directed speech: relation to socioeconomic status, knowledge of child development and child vocabulary skill. J. Child Lang. **35**, 185–205. (10.1017/s0305000907008343) 18300434

[73] Schieffelin B, Ochs E (eds). 1986 Language socialization across cultures. Cambridge, UK: Cambridge University Press.

[74] Abels M, Kilale A, Vogt P. 2020 Speech acts addressed to Hadza infants in Tanzania: cross-cultural comparison, speaker age, and camp livelihood. First Lang. **41**, 294–313. (10.1177/0142723720972000)

[75] Keller H *et al*. 2006 Cultural models, socialization goals, and parenting ethnotheories. J. Cross Cult. Psychol. **37**, 155–172. (10.1177/0022022105284494)

[76] Lamm B, Keller H. 2009 Understanding cultural models of parenting: the role of intracultural variation and response style. J. Cross Cult. Psychol. **38**, 50–57. (10.1177/0022022106295441)

[77] Vogt P, Mastin JD, Schots DMA. 2015 Communicative intentions of child-directed speech in three different learning environments: observations from the Netherlands, and rural and urban Mozambique. First Lang. **35**, 341–358. (10.1177/0142723715596647)

[78] Rabain-Jamin J. 2001 Language use in mother-child and young sibling interactions in Senegal. First Lang. **21**, 357–385. (10.1177/014272370102106307)

[79] Pye C. 1986 Quiché Mayan speech to children. J. Child Lang. **13**, 85–100. (10.1017/s0305000900000313) 3949901

[80] Moore LC. 2011 Language socialization and repetition. In The handbook of language socialization (eds A Duranti, E Ochs, B Schieffelin), pp. 209–226. Oxford, UK: Wiley-Blackwell. (10.1002/9781444342901)

[81] Fey ME, Krulik TE, Loeb DF, Proctor-Williams K. 1999 Sentence recast use by parents of children with typical language and children with specific language impairment. Am. J. Speech Lang. Pathol. **8**, 273–286. (10.1044/1058-0360.0803.273)

[82] Taumoepeau M. 2016 Maternal expansions of child language relate to growth in children’s vocabulary. Lang. Learn. Dev. **12**, 429–446. (10.1080/15475441.2016.1158112)

[83] Rogoff B, Mistry J, Göncü A, Mosier CE, Schieffelin B. 1993 Guided participation in cultural activity by toddlers and caregivers. Monogr. Soc. Res. Child Dev **58**, 1–174. (10.2307/1166109) 8284000

[84] Legare CH. 2017 Cumulative cultural learning: development and diversity. Proc. Natl Acad. Sci. USA **114**, 7877–7883. (10.1073/pnas.1620743114) 28739945 PMC5544271

[85] Kabha L, Berger A. 2020 The sequence of acquisition for theory of mind concepts: the combined effect of both cultural and environmental factors. Cogn. Dev. **54**, 100852. (10.1016/j.cogdev.2020.100852)

[86] Kuntoro IA, Peterson CC, Slaughter V. 2017 Culture, parenting, and children’s theory of mind development in Indonesia. J. Cross Cult. Psychol. **48**, 1389–1409. (10.1177/0022022117725404)

[87] Laranjo J, Bernier A, Meins E, Carlson SM. 2010 Early manifestations of children’s theory of mind: the roles of maternal mind‐mindedness and infant security of attachment. Infancy **15**, 300–323. (10.1111/j.1532-7078.2009.00014.x) 32693541

[88] Meins E, Fernyhough C, Wainwright R, Clark-Carter D, Das Gupta M, Fradley E, Tuckey M. 2003 Pathways to understanding mind: construct validity and predictive validity of maternal mind-mindedness. Child Dev. **74**, 1194–1211. (10.1111/1467-8624.00601) 12938713

[89] Hughes C, Devine RT, Wang J. 2017 Does parental mind‐mindedness account for cross‐cultural differences in preschoolers’ theory of mind? Child Dev. **89**, 1296–1310. (10.1111/cdev.12746) 28160284 PMC6849528

[90] Chan MH, Wang Z, Devine RT, Hughes C. 2020 Parental mental-state talk and false belief understanding in Hong Kong children. Cogn. Dev. **55**, 100926. (10.1016/j.cogdev.2020.100926)

[91] Naito M, Koyama K. 2006 The development of false-belief understanding in Japanese children: delay and difference? Int. J. Behav. Dev. **30**, 290–304. (10.1177/0165025406063622)

[92] Liu Y, Wang Y, Luo R, Su Y. 2016 From the external to the internal: behavior clarifications facilitate theory of mind (ToM) development in Chinese children. Int. J. Behav. Dev. **40**, 21–30. (10.1177/0165025414562484)

[93] Lavelle JS. 2021 The impact of culture on mindreading. Synthese **198**, 6351–6374. (10.1007/s11229-019-02466-5)

[94] Selcuk B, Gonultas S, Ekerim‐Akbulut M. 2022 Development and use of theory of mind in social and cultural context. Child Dev. Perspect. **17**, 39–45. (10.1111/cdep.12473)

[95] Dixson HGW, Komugabe‐Dixson AF, Dixson BJ, Low J. 2018 Scaling theory of mind in a small‐scale society: a case study from Vanuatu. Child Dev. **89**, 2157–2175. (10.1111/cdev.12919) 28984351

[96] Shahaeian A, Peterson CC, Slaughter V, Wellman HM. 2011 Culture and the sequence of steps in theory of mind development. Dev. Psychol. **47**, 1239–1247. (10.1037/a0023899) 21639620

[97] Taumoepeau M, Kata UF, Veikune AH, Lotulelei S, Vea PT, Fonua I. 2022 Could, would, should: theory of mind and deontic reasoning in Tongan children. Child Dev. **93**, 1511–1526. (10.1111/cdev.13797) 35616232 PMC9545884

[98] Wellman HM, Fang F, Liu D, Zhu L, Liu G. 2006 Scaling of theory-of-mind understandings in Chinese children. Psychol. Sci. **17**, 1075–1081. (10.1111/j.1467-9280.2006.01830.x) 17201790

[99] Wellman HM, Liu D. 2004 Scaling of theory‐of‐mind tasks. Child Dev. **75**, 523–541. (10.1111/j.1467-8624.2004.00691.x) 15056204

